# Tsantan Sumtang Alleviates Chronic Hypoxia-Induced Pulmonary Hypertension by Inhibiting Proliferation of Pulmonary Vascular Cells

**DOI:** 10.1155/2018/9504158

**Published:** 2018-11-28

**Authors:** Qilian He, Xingmei Nan, Silin Li, Shanshan Su, Ke Ma, Zhanqiang Li, Dianxiang Lu, Rili Ge

**Affiliations:** ^1^Research Center for High Altitude Medicine, Qinghai University, Xining 810001, China; ^2^Key Laboratory of Application and Foundation for High Altitude Medicine Research in Qinghai Province, Xining 810001, China; ^3^Medical Department, Medical College, Qinghai University, Xining 810001, China; ^4^Affiliated Hospital of Heilongjiang University of Traditional Chinese Medicine, Ha'erbin 150040, China; ^5^Qinghai Entry-Exit Inspection and Quarantine Bureau, Xining 810003, China

## Abstract

Hypoxia-induced pulmonary hypertension (HPH) is a severe condition associated with significant morbidity and mortality in people living at high altitude. Tsantan Sumtang, a traditional Tibetan medicine, has been routinely used for the treatment of cardiopyretic disease, as well as stenocardia. Interestingly, our previous research found that Tsantan Sumtang improved HPH in rats maintaining in a hypobaric chamber. We performed a series of experiments to test the indexes of vasoconstriction and vascular remodeling, the key pathophysiological characteristics of HPH. Our results showed that Tsantan Sumtang relaxed noradrenaline (NE)-precontracted rat pulmonary artery rings in a concentration-dependent manner* in vitro*. The PGI2-cAMP (prostaglandin I2-cyclic adenosine monophosphate) pathway, NO-cGMP (nitric oxide-cyclic guanosine monophosphate) pathway, and the opening of K^+^ channels (inward rectifier K^+^ channels, large conductance Ca^2+^-activated K^+^ channels, and voltage-dependent K^+^ channels) might play major roles in the vasorelaxation effect.* In vivo*, the administration of Tsantan Sumtang resulted in a substantial decrease in the rat mean pulmonary artery pressure (mPAP) and the right ventricular hypertrophy index (RVHI). The reduction of thickness of small pulmonary arterial wall and the WT% (the ratio of the vascular wall thickness to the vascular diameter) were observed. The smooth muscle muscularization of the arterials was alleviated by Tsantan Sumtang treatment at the same time. Tsantan Sumtang also reduced remodeling of pulmonary arterioles by suppressing the expression of proliferating cell nuclear antigen (PCNA), *α*-smooth muscle actin (*α*-SMA), cyclin D1, and cyclin-dependent kinase 4 (CDK4) through inhibition of p27Kip1 degradation. Therefore, Tsantan Sumtang could be applied as a preventative medication for HPH, which would be a new use for this traditional medicine.

## 1. Introduction

Pulmonary hypertension (PH) is a group of clinical disorders that feature an abnormal elevation in the pulmonary circulatory pressure and are characterized by a sustained increase in pulmonary vascular resistance and vascular remodeling, which ultimately leads to right ventricular failure and death [1, 2].

HPH is a type of PH that occurs among residents and travelers at high altitude. It has been reported that people living on a plateau may suffer from minor to moderate pulmonary vasoconstriction and hypertension, and those with a maladaptation to hypoxia may induce hypoxia-related diseases, among which HPH appears in approximately 5–10% of cases [3–5]. Sustained pulmonary vasoconstriction and structural remodeling of small pulmonary arteries result in right ventricular hypertrophy and heart failure. HPH is associated with significant morbidity and mortality, but a specific and effective therapy remains unavailable [6–8]. As the key pathophysiological changes with HPH, vasoconstriction and vascular remodeling of the pulmonary artery impact the long-term prognosis of the patient, especially regarding myocardial damage, right ventricular hypertrophy, heart failure, and brain function decline [1, 2].

Tibetan medicament is a compound medicine with a long history of treatment effectiveness. This treatment approach attempts to harmonize the body, mind, and spirit [9]. The inclusive and flexible remedy strategies of Tibetan medicine always generate a prominent cure result based on herbal medication, and an increasing number of people in the world have become interested in Tibetan medicine. Tsantan Sumtang is recorded in the Tibetan medical classics* Four Tantras* as a traditional cardiovascular therapeutic prescription and is composed of* Choerospondias axillaris *(Roxb.) Burtt et Hill*, Santalum album *L., and* Myristica fragrans *Houtt [10]. Tsantan Sumtang is traditionally decocted as a water solution for the treatment of cardiovascular disease, which is similar to stenocardia. Interestingly, our previous research found that Tsantan Sumtang could significantly attenuate the increase in the mean pulmonary artery pressure (mPAP) and decrease the right ventricular hypertrophy index (RVHI) in a HPH rat model established in a hypobaric cabin that simulates a high altitude of 4500 m. However, the mechanism is still unclear [11].

Therefore, based on the above background and our previous research results, this study aimed to explore the effects and the underlying mechanisms of Tsantan Sumtang in pulmonary artery vasoconstriction and remodeling in HPH rats to provide evidence for a potential new clinical use of this traditional Tibetan medicine.

## 2. Materials and Methods

### 2.1. Medicine, Reagents, and Instruments

Tsantan Sumtang powder composed of* C. axillaris *(Roxb.) Burtt et Hill (100 g),* S. album *L. (100 g), and* M. fragrans *Houtt (100 g) was purchased from Qinghai Tibetan Medicine Hospital (Xining, Qinghai, China), the institute of authority in the area of Tibetan medicine. Noradrenaline (NE, catalog # H12020621) and acetylcholine (ACh, ≥98%, catalog # 60-31-1) were purchased from Jin Yao Amino Acid Co., Ltd. (Tianjin, China). Indomethacin (IMT, catalog # 182810), N*ω*-nitro-L-arginine methyl ester hydrochloride (L-NAME, catalog # 101150336), tetraethyl ammonium chloride (TEA, catalog # 28460), barium chloride (BaCl_2_, catalog # 1001900129), and 4-aminopyridine (4-AP, catalog # 275875) were purchased from Sigma Chemical Co. (St. Louis, MO, USA); anti-PCNA (Antibody catalog # ab29), anti-*α*-SMA (Antibody catalog # ab7817), anti-CDK4 (Antibody catalog # AB199728), anti-cyclin D1 (Antibody catalog # ab134175), anti-p27Kip1 (Antibody catalog # ab193379), and anti-*β*-actin antibodies (Antibody catalog # ab8226) were purchased from Abcam Biotechnology (Cambridge, MA, USA). Primers for CDK4, cyclin D1, p27Kip1, and *β*-Actin were provided by the QIAGEN Company (QIAGEN China Co., Ltd., Shanghai, China). The following instruments were used: a rotary evaporator (RE-52, Ya Rong Biochemical Instrument Factory, Shanghai, China), force transducer (JH-2, Space Medico-Engineering Institute, Beijing, China), biological function experimental system (BL-420, Tai Meng Technology Co., Ltd., Chengdu, China), and* in vitro* tissue and organ perfusion system (HV-4, Tai Meng Technology Co., Ltd., Chengdu, China). An automatically adjusted low-pressure hypobaric chamber (DYC-300, Guizhou Fenglei Oxygen Chamber Co., Ltd., Guizhou, China) was used in constructing the HPH animal model and Data Acquisition System (MP100, Biopac Systems Inc., CA, USA) was used for pulmonary pressure measurement.

### 2.2. Animals

The experimental protocol was approved by the Institutional Animal Care and Use Committee of Qinghai University in compliance with the animal management rules of the Chinese Ministry of Health. Specific pathogen-free (SPF) grade male Sprague Dawley (SD) rats weighing 250 ± 20 g were supplied by the Animal Center of Xi'an Jiaotong University, China. The permit number is SCXK(Shan)2012-003. The animals were fed a standard pelleted diet* ad libitum*, and water was available without restriction at an ambient temperature of 22 ± 2°C and relative humidity of 45%–55% throughout the experiments. All efforts were made to reduce animal distress by controlling factors and to limit the number of rats used for experimentation.

### 2.3. *In Vitro* Pulmonary Artery Perfusion Experiments

#### 2.3.1. Preparation of an Aqueous Extract of Tsantan Sumtang

Tsantan Sumtang, 30.0 g in a 500 mL volumetric flask with 150 mL water, was reflux-extracted 3 times, for 60 minutes each time. The extracted solution was collected for rotary evaporation, concentrated to a paste, and then diluted with deionized water to 400 mL; the material was kept in a freezer at −20°C in a separate package.

#### 2.3.2. Preparation of Rat Pulmonary Arterial Rings

Rats were anesthetized with urethane (1.0 g/kg) by intraperitoneal injection, and their hearts and lungs were then dissected and instantly submerged in an ice-cold KH solution composed of the following ingredients (in mmol/L): 118.0 NaCl, 4.7 KCl, 2.5 CaCl_2_, 1.2 MgSO_4_,·7 H_2_O, 1.2 KH_2_PO_4_, 25 NaHCO_3_, and 11.1 glucose (pH 7.4). The intrapulmonary arterm was separated free from close connective tissue and then cut into vessel rings of approximately 2-3 mm in length. The rings were suspended in organ chambers filled with 10 mL of KH solution at 37°C and gassed with 95% O_2_ and 5% CO_2_; isometric tension was measured using force transducer and biological function experimental system.

#### 2.3.3. Assessment of Vessel Ring Activity and Endothelial Function

Arterial rings were equilibrated for 2 hours under a basic pressure of 400 mg while the KH solution was changed every 15 minutes. Next, 1 *μ*mol/L of NE was added into the chamber to determine the percentage of vessel contraction. The vessels with a contraction percentage under 100% were not used as samples. The integrity was measured as the rate of relaxation produced by ACh (10 *μ*mol/L) from the percentage of contraction induced by NE (1 *μ*mol/L). A relaxation of more than 80% by ACh indicated that the endothelium remained in a good state, while rings with less than 80% relaxation were excluded as samples [12].

### 2.4. Effect and Mechanism of Tsantan Sumtang on NE-Precontracted Pulmonary Artery* In Vitro*

The effective dosage of Tsantan Sumtang was explored in our study and was finally determined to be 0.3-1.2 mg/mL at every 0.3 mg/mL interval. After vasoconstriction of the intact endothelium vessel rings caused by 1 *μ*mol/L NE reached a plateau (simulating the vasoconstriction in HPH), aqueous extracts of Tsantan Sumtang from 0.3 to 1.2 mg/mL were cumulatively added. To determine the underlying mechanisms involved in Tsantan Sumtang-induced relaxation, different inhibitors, including L-NAME (100 *μ*mol/L), IMT (10 *μ*mol/L), TEA (1 mmol/L), BaCl_2_ (1 mmol/L), and 4-AP (1 mmol/L), were individually added after the 1 *μ*mol/L NE-induced vessel ring vasoconstriction reached a plateau; then, from 0.3 to 1.2 mg/mL of Tsantan Sumtang was added, cumulatively, after the new vasoconstriction reached a plateau. The same volume of KH solution was added to the control chamber in both experiments.

### 2.5. Effect and Mechanism of Tsantan Sumtang on HPH Rats* In Vivo*

#### 2.5.1. Groups, Medicine Preparation, and HPH Animal Model Establishment

Rats were randomly separated into five groups: a control group, a hypoxia group (rats were placed in a hypobaric chamber for 4 weeks with the inner pressure and oxygen content concomitantly adjusted to be equal to those at an altitude of 4500 m), and hypoxia + Tsantan Sumtang treatment groups. We spent approximately 1 hour every day administering medicine, feeding, watering, and cleaning up. On the 28th day, the mPAP and RVHI were measured to detect the possible effect of Tsantan Sumtang and to determine whether the HPH model rats were successfully established.

The recommended dosage of Tsantan Sumtang for adults is 6-9 g per day. The equivalent dose in rats is approximately 10 times the human dose. Hence, we chose 1.0 g/kg (equal to 6 g for an adult), 1.25 g/kg (equal to 7.5 g for an adult), and 1.5 g/kg (equal to 9 g for an adult) as low, middle, and high dosages, respectively. Because Tsantan Sumtang powder is traditionally used in a solution, the medicine was decocted with water 3 times, for 15 minutes each time and the mixed decoctions were then adjusted to the needed concentrations and administered by gavage to the hypoxia + Tsantan Sumtang groups, while the same volume of 0.9% normal saline was given to the hypoxia group during the 4 weeks.

For the mPAP measurement, a polystyrene microcatheter thoroughly soaked with heparin saline was used to connect the pressure transducer to the Biopac MP100 Data Acquisition System. After urethane (1.0 g/kg) was intraperitoneally injected based on the rat weight, the right external jugular vein was carefully shunted, and the catheter was slowly pushed proximally; the location of the catheter was then determined based on the waveform until a typical pulmonary artery pressure waveform appeared. After a stabilization period of 4 minutes, the mean pulmonary artery pressure was recorded. The RVHI, the ratio of the right ventricular free wall (RV) to the left ventricle (LV) plus the interventricular septum (S), was calculated as RV/(LV+S) after drying the two parts with filter paper and weighing them separately.

#### 2.5.2. Morphometric Evaluation of Lung Tissue

After the mPAP and RVHI measurements, the rats were sacrificed and the left lung tissues were fixed in 4% paraformaldehyde solution for 48 hours. The tissues were dehydrated and embedded in paraffin. Sections of 5 *μ*m thick were prepared and stained with hematoxylin and eosin (HE) for histopathological evaluation. Morphological changes were observed with a BA400 Digital microscope (Motic China Group Co., Ltd., Chengdu, China) and measured by Image-Pro Plus 6.0 software (Media Cybernetics, Rockville, Maryland, USA). Ultrastructural changes in small pulmonary arteries were examined by means of transmission electron microscopy (TEM). The samples were immersion-fixed with 3% buffered glutaraldehyde. After fixation, the tissues were stored in glutaraldehyde in a refrigerator overnight. The tissue blocks were rinsed in 0.1 M phosphate buffer and post-fixed for 2 h with 1% osmium tetroxide in 0.125 M sodium cacodylate buffer. They were then dehydrated in increasing concentrations (30-100%) of ethanol, rinsed in acetone, and embedded in Araldite. Ultrathin-sections (50 nm) were cut and stained with uranyl acetate and lead citrate and examined with a Hitachi H-600 IV electron microscope (Hitachi High Technologies Co., Ltd., Shanghai, China).

### 2.6. Western Blot Analysis

The protein expression levels of PCNA, *α*-SMA, CDK4, cyclin D1, and p27Kip1 in lung tissue were determined by Western blot analysis. The frozen lung tissue was homogenized in Radio Immunoprecipitation Assay (RIPA) buffer and centrifuged at 10,000 g for 15 minutes at a temperature of 4°C. After centrifuging, the protein concentration of the supernatant was measured with the Pierce™ BCA Protein Assay Kit supplied by Beyotime Institute of Biotechnology (Shanghai, China) with bovine serum albumin as the standard sample. The proteins (50 *μ*g/lane) were separated using sodium dodecyl sulfate–polyacrylamide gel electrophoresis (SDS-PAGE) and transferred to a polyvinylidene difluoride membrane. The membrane was blocked with TBST containing 5% nonfat dry milk and incubated with anti-PCNA antibodies, anti-*α*-SMA antibodies, anti-CDK4 antibodies, anti-cyclin D1 antibodies, anti-p27Kip1 antibodies, and anti-*β*-actin antibodies at a concentration of 1:2500 (PCNA, *α*-SMA, CDK4, p27Kip1) or 1:10000 (cyclin D1), overnight at 4°C. The membrane was then incubated with goat anti-mouse/anti-rabbit immunoglobulin G at a concentration of 1:5000 and subsequently visualized with an enhanced chemiluminescence (ECL) kit (Biyuntian Biotech Institute, Beijing, China). Equal lane loading was assessed using *β*-actin.

### 2.7. Real-Time Quantitative Polymerase Chain Reaction (RT-qPCR)

A TRIzol reagent was used to extract total RNA from the lung tissue. cDNA was then prepared from 2 *μ*g of purified RNA using a Takara PrimeScript RT reagent kit with gDNA Eraser according to the manufacturer's protocol. The mRNA expression of CDK4, cyclin D1, and p27Kip1 was determined by Takara TB Green Premix Ex Taq with ABI 7500 Real-Time PCR system (Bio-Rad, CA, USA). The PCR conditions were as follows: 95°C for 30 seconds, and 40 cycles of 95°C for 5 seconds, 60°C for 34 seconds, followed by a melting curve analysis (95°C for 15 seconds, 60°C for 1 minute, and then 95°C for 30 seconds and 60°C for 15 seconds). Experimental genes were normalized to *β*-actin. Primers were amplified with equal efficiencies. The 2^−ΔΔct^ method was used to analyze the results.

### 2.8. Statistical Analysis

The results are expressed as the mean ± SD. Statistical analysis was performed by means of one-way analysis of variance (ANOVA), followed by Student-Newman-Keuls test and Dunnett's multiple comparison test. Significance was defined as* P*≤0.05.

## 3. Results

### 3.1. The Vasorelaxation Effect and Mechanism of Tsantan Sumtang on Pulmonary Arteries* In Vitro*

**Figure 1:**
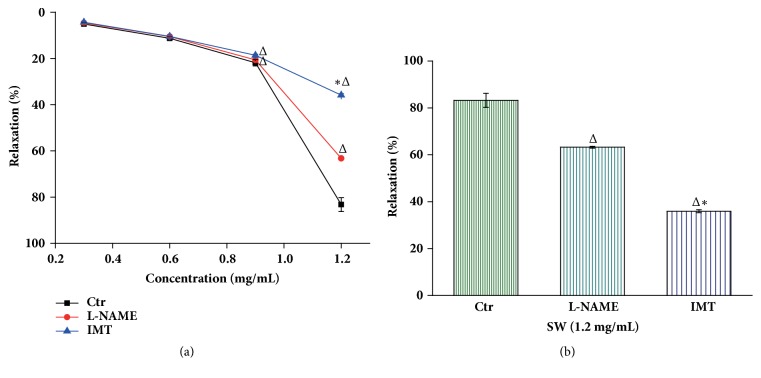
**The vasorelaxation effect of Tsantan Sumtang on intact endothelium pulmonary arterial rings and the effects of L-NAME and IMT on Tsantan Sumtang induced rat pulmonary artery vasorelaxation (n=6).** Ctr: control group administered with Tsantan Sumtang but without any inhibitors. SW: Tsantan Sumtang. Tsantan Sumtang dilated the vessels gradually from 0.3 to 0.9 mg/mL and roared to the max relaxation at 1.2 mg/mL. To test the mechanisms involved in the vasorelaxation effect of Tsantan Sumtang, different inhibitors were used. The rings were constricted furthest with 1 *μ*mol/L of NE, and then L-NAME (eNOS inhibitor, 100 *μ*mol/L) and IMT (cyclooxygenase inhibitor, 10 *μ*mol/L) were administered. After reaching a new stable phase, Tsantan Sumtang was added in an accumulative way (from 0.3 to 1.2 mg/mL). The dosage reactions with or without the inhibitors were shown as (a) and column (b) giving a more visual image for the max relaxation rates (^Δ^*P*<0.05 compared to control group, ^*∗*^*P<0.05* compared to L-NAME).

**Figure 2:**
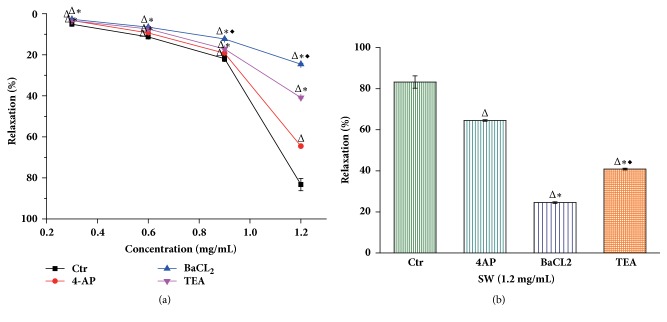
**The influences of K** ^**+**^ **channels inhibitors on Tsantan Sumtang-induced rat pulmonary artery vasorelaxation (n=6).** Ctr: control group administered with Tsantan Sumtang but without the inhibitors. SW: Tsantan Sumtang. TEA (large-conductance Ca^2+^-activated K^+^ channel inhibitor, 1 mmol/L), BaCl_2_ (inward rectifier K^+^ channel inhibitor,1 mmol/L), and 4-AP (voltage-dependent K^+^ channel inhibitor, 1 mmol/L) were used to test the mechanisms involved in Tsantan Sumtang-induced rat pulmonary artery vasorelaxation too. The dosage reactions with or without the inhibitors were shown as (a) and column (b) giving a more visual image for the max relaxation rates. The vasorelaxation effect of Tsantan Sumtang was markedly blocked by all three K^+^ channel inhibitors, with a max reduction to 64.5%  ± 0.38%, 24.56%  ± 0.38%, and 40.87%  ± 0.40%, respectively (^Δ^*P*<0.05 compared to control group). Among them, BaCl_2_ and TEA expressed stronger blocking effect than 4-AP (^*∗*^*P<0.05* compared to 4-AP) and BaCl_2_ is the strongest blocker (^◆^*P<0.05* compared to TEA).

After the NE-induced vessel ring constriction reached a plateau, increasing doses of Tsantan Sumtang were added (0.3-1.2 mg/mL) to the rings with an intact endothelium, which gradually dilated the vessels, reaching a maximum of 83.25±3.00% at 1.2 mg/mL. Different doses of Tsantan Sumtang had dose-related vasorelaxation effects as shown in the control group, and the relaxation rates with 0.3 mg/mL, 0.6 mg/mL, and 0.9 mg/mL of Tsantan Sumtang were 5.12±0.74%, 11.28±0.75%, and 21.89±1.35%, respectively, but the relaxant effect plateaued at a maximum of 83.25±3.00% with 1.2 mg/mL even when we added more Tsantan Sumtang (Figures 1(a) and 2(a)). Next, after the different inhibitors were added, we found that the vasorelaxation caused by the Tsantan Sumtang treatment of small pulmonary artery rings that had been contracted with 1 *μ*mol/L NE was significantly blocked by L-NAME (an eNOS inhibitor, 100 *μ*mol/L), IMT (a cyclooxygenase inhibitor, 10 *μ*mol/L), TEA (a large-conductance Ca^2+^-activated K^+^ channel inhibitor, 1 mmol/L), BaCl_2_ (an inward rectifier K^+^ channel inhibitor, 1 mmol/L), and 4-AP (a voltage-dependent K^+^ channel inhibitor, 1 mmol/L) with maximum contractions of 63.23±0.37%, 35.90±0.64%, 64.50±0.38%, 24.56±0.38%, and 40.87±0.40%, respectively, (*P*<0.05 versus the control group of 83.25±3.00%, Figures 1 and 2). In detail, the vasorelaxation by Tsantan Sumtang was inhibited by L-NAME (100 *μ*mol/L) and IMT (10 *μ*mol/L) at both the 0.9 and 1.2 mg/mL levels, but IMT had a greater inhibitory effect than L-NAME at 1.2 mg/mL of Tsantan Sumtang level (*P*<0.05, Figures 1(a) and 1(b)). Additionally, the pulmonary artery vasorelaxation at all 4 doses was also inhibited by the K^+^ channel inhibitors 4-AP, BaCl_2_, and TEA (all at 1 mmol/L) (*P*<0.05, Figures 2(a) and 2(b)). Compared to 4-AP, BaCl_2_ and TEA showed stronger inhibitory effects at all 4 dose levels, while BaCl_2_ performed better than TEA at the 0.9 mg/mL and 1.2 mg/mL levels (*P<0.05*, Figures 2(a) and 2(b)). The differences in the maximum effect of all the inhibitors at the high dose of 1.2 mg/mL can be visualized in greater detail in Figures 1(b) and 2(b).

### 3.2. Effects and Mechanisms of Tsantan Sumtang on HPH Rats* In Vivo*

#### 3.2.1. Tsantan Sumtang Decreased the Rise of the mPAP in HPH Model Rats

**Figure 3:**
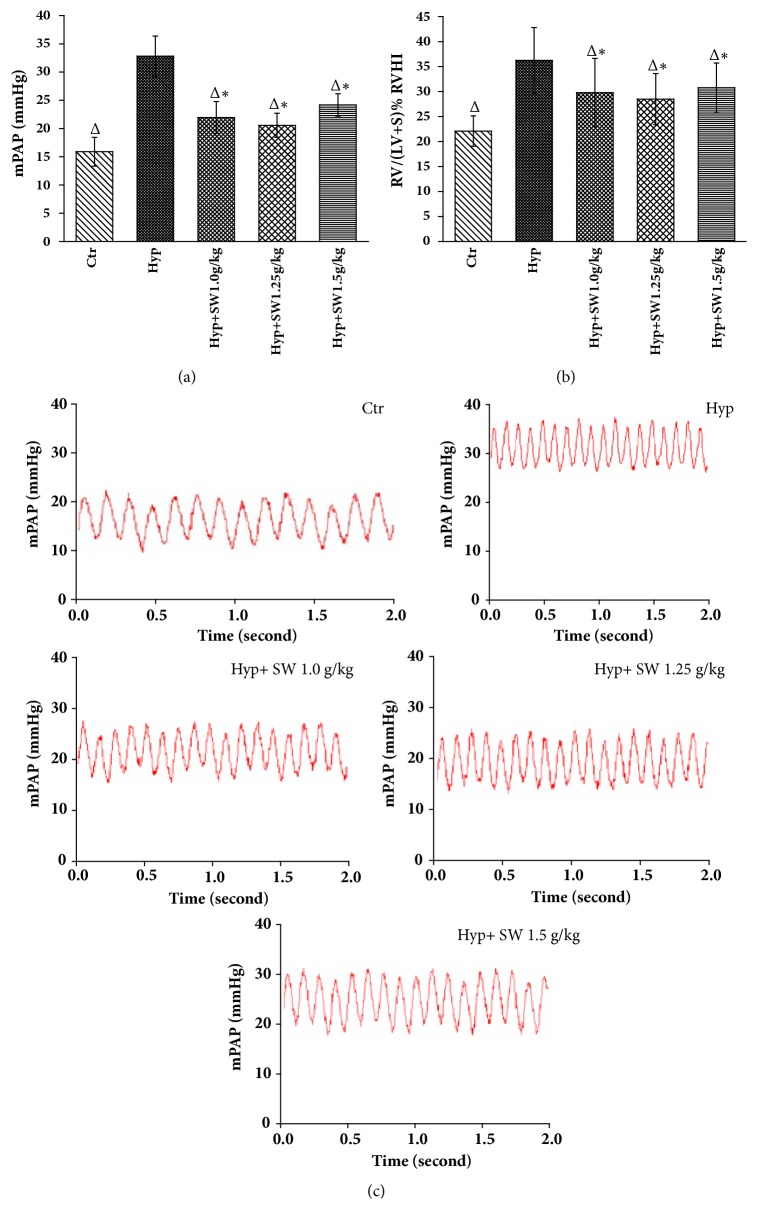
**Chronic HPH model construction and alleviation effect caused by Tsantan Sumtang during 4 weeks (n=6).** Ctr: control group, Hyp: untreated hypoxia group (rats exposed to hypoxia in hypobaric chamber, equal to the parameter in altitude 4500 m), and Hyp + SW-treated groups (rats exposed to hypoxia in hypobaric chamber and received increasing doses of Tsantan Sumtang treatment, 1.0 g/kg, 1.25 g/kg, and 1.5 g/kg). (a, b) mPAP: mean pulmonary arterial pressure and RVHI: right ventricular hypertrophy index. Both mPAP and RVHI were much higher in Hyp group and all 3 levels of Tsantan Sumtang treatment decreased mPAP and RVHI significantly but still had difference with the control rats (^Δ^*P*< 0.05 compared to Hyp; ^*∗*^*P*< 0.05 compared to Ctr). (c) Representative pictures of mPAP waves in Ctr, Hyp, and Hyp+SW-treated groups.

On day 28, the mPAP and RVHI in the hypoxia group increased to 32.81±3.58 mmHg and 36.23±6.58%, respectively, compared with 15.89 ± 2.54 mmHg and 22.09±3.04% in the control group, proving that the HPH model rats were successfully established (*P<0.05*, Figures 3(a)–3(c)). After treatment with Tsantan Sumtang, both the mPAP and RVHI decreased significantly in all 3 dosage groups compared to the hypoxia group and decreased greatly in the middle-dosage group (*P*< 0.05, Figures 3(a) and 3(b)).

#### 3.2.2. Tsantan Sumtang Alleviated the Morphological and Ultrastructural Changes in Small Pulmonary Arteries of HPH Rats

**Figure 4:**
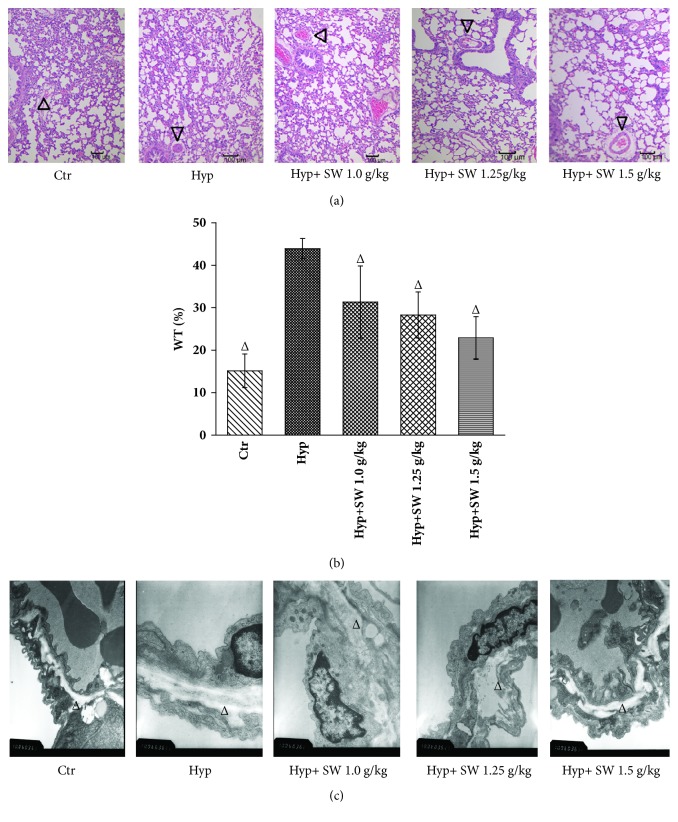
**Tsantan Sumtang alleviated chronic hypoxia-induced pulmonary vascular remodeling in 4 weeks (n=5).** Ctr: control group; Hyp: untreated hypoxia group (rats exposed to hypoxia in hypobaric chamber, equal to the parameter in altitude 4500 m), and Hyp+SW-treated groups (rats exposed to hypoxia in hypobaric chamber and received increasing doses of Tsantan Sumtang treatment, 1.0 g/kg, 1.25 g/kg, and 1.5 g/kg). (a) HE staining of the small pulmonary arterioles (diameters below100 *μ*m, ×100). Triangle indicated that pulmonary small vessels thickened in Hyp rats and were lessened notably by Tsantan Sumtang treatment. (b) The ratio of the vascular wall thickness to the vascular diameter (WT%) of pulmonary arterioles. WT% was increased obviously in Hyp group and alleviated markedly by Tsantan Sumtang treatment (^Δ^*P*< 0.05 compared to untreated hypoxic rats). (c) Ultrastructure of pulmonary arterioles by transmission electron microscope (TEM, ×20000) which indicated smooth muscle muscularization of the arterial vascular wall, especially in the tunica media induced by chronic hypoxia. Tsantan Sumtang treatment groups showed obvious alleviation of tunica media thickening (triangle indicated the changes of tunica media in pulmonary arterial vascular wall).

The morphology and ultrastructure of the walls of small pulmonary arteries were observed by HE staining and TEM, as reported in other experiments [13, 14]. The HE staining indicated evenly distributed endothelial cells within the thin and continuous arteriole wall of the control group. In the HPH group, both the HE staining and TEM revealed thicker arterial vessel walls and decreased lumen diameters (Figures 4(a) and 4(c)), consistent with the results reported in previous papers [15–18]. The Tsantan Sumtang group exhibited less vessel wall thickness, especially in the tunica media layer, than the HPH group (Figures 4(a) and 4(c)). The WT% increased to 43.92±2.37% in the hypoxia group compared with 15.15±3.97% in the control group (*P<0.05,*Figure 4(b)). The Tsantan Sumtang treatment groups showed significant alleviating effects on the WT% in the HPH rats, even though there was difference between these rats and the normal rats (*P*< 0.05, Figure 4(b)).

#### 3.2.3. PCNA and *α*-SMA Protein Expression after Tsantan Sumtang Treatment

**Figure 5:**
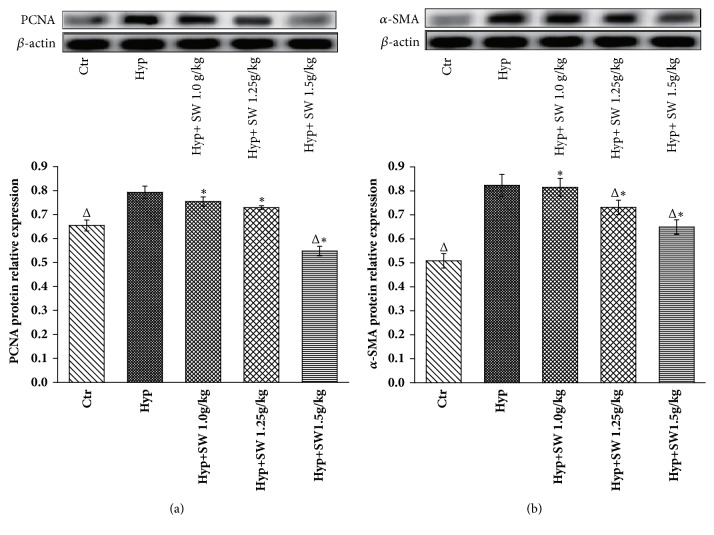
**Effects of Tsantan Sumtang on PCNA and **α**-SMA protein expression in lung tissue of HPH rats (n=5). **Ctr: control group; Hyp: untreated hypoxia group (rats exposed to hypoxia in hypobaric chamber, equal to the parameter in altitude 4500 m), and Hyp+SW-treated groups (rats exposed to hypoxia in hypobaric chamber and received increasing doses of Tsantan Sumtang treatment, 1.0 g/kg, 1.25 g/kg, and 1.5 g/kg). Upper panel: relative expression levels of PCNA (a) and *α*-SMA (b) protein (optical density of PCNA and *α*-SMA normalized against *β*-actin expression). Lower panel: *β*-actin protein expression was used as control. (a) PCNA expression was significantly higher in Hyp group and Tsantan Sumtang treatment of 1.5 g/kg significantly reduced protein expression of PCNA in HPH rats, even below the control rats (^Δ^*P*< 0.05 compared to Hyp; ^*∗*^*P*< 0.05 compared to Ctr); (b) *α*-SMA expression was higher in Hyp group and Tsantan Sumtang treatment of 1.25 g/kg and 1.5 g/kg significantly downregulated protein expression of *α*-SMA in HPH rats but still higher than the control rats (^Δ^*P*< 0.05 compared to Hyp; ^*∗*^*P*< 0.05 compared to Ctr).

Western blot analysis was used to identify the pulmonary vascular antiproliferative mechanism involved in Tsantan Sumtang treatment that could substantiate the effects shown above. The results indicated that the protein expression levels of PCNA and *α*-SMA in the hypoxia groups were both noticeably higher than those in the control groups (*P*<0.05, Figure 5). The high-dosage Tsantan Sumtang group showed differences in PCNA expression compared to the HPH rats and meaningfully downregulated PCNA expression to below the level of the control group (*P*< 0.05, Figure 5(a)). The middle and high dosages had meaningful alleviating effects on *α*-SMA expression compared to that in the HPH rats, but there was still difference in comparison to the level in the control rats (Figure 5(b)).

#### 3.2.4. Cyclin D1, CDK4, and p27Kip1 Protein and Gene Expression Levels after Tsantan Sumtang Treatment

**Figure 6:**
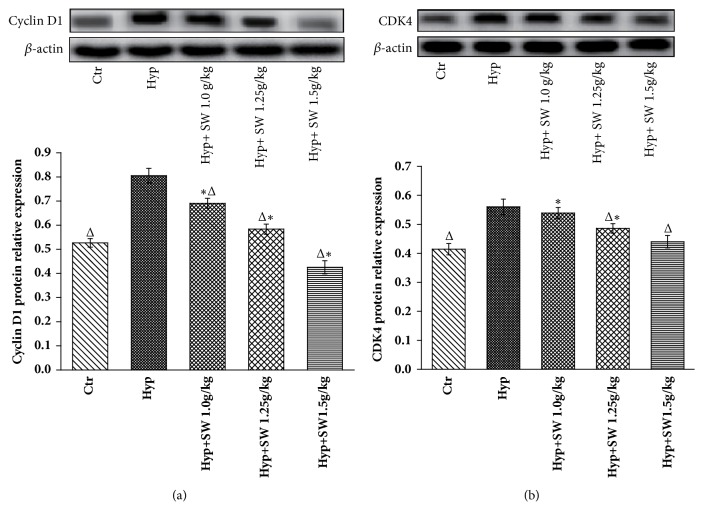
**Effects of Tsantan Sumtang on Cyclin D1 and CDK4 protein expression in lung tissue of HPH rats (n=5).** Ctr: control group; Hyp: untreated hypoxia group (rats exposed to hypoxia in hypobaric chamber, equal to the parameter in altitude 4500 m), and Hyp+SW-treated groups (rats exposed to hypoxia in hypobaric chamber and received increasing doses of Tsantan Sumtang treatment, 1.0 g/kg, 1.25 g/kg, and 1.5 g/kg). Upper panel: relative expression levels of Cyclin D1 (a) and CDK4 (b) protein (optical density of Cyclin D1 and CDK4 normalized against *β*-actin expression). Lower panel: *β*-actin protein expression was used as control. (a) Cyclin D1 expression was significantly higher in Hyp group and Tsantan Sumtang treatment of all 3 doses significantly downregulated protein expression of Cyclin D1 in HPH rats with the best effect of high dose (^Δ^*P*< 0.05 compared to Hyp; ^*∗*^*P*< 0.05 compared to Ctr); (b) CDK4 expression was meaningfully higher in Hyp group and Tsantan Sumtang treatment of 1.25 g/kg and 1.5 g/kg doses considerably downregulated protein expression of CDK4 in HPH rats, and the 1.5 g/kg Tsantan Sumtang treatment group showed best effect with no difference with the control rats on CDK4 expression (^Δ^*P*< 0.05 compared to Hyp; ^*∗*^*P*< 0.05 compared to Ctr).

**Figure 7:**
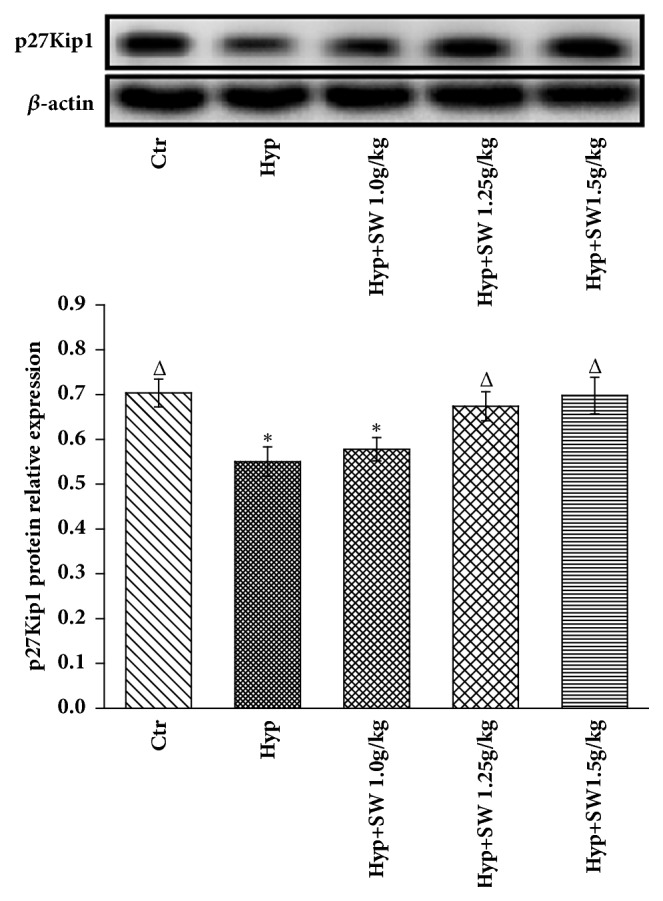
**Effects of Tsantan Sumtang on p27Kip1 protein expression in lung tissue of HPH rats (n=5).** Ctr: control group; Hyp: untreated hypoxia group (rats exposed to hypoxia in hypobaric chamber, equal to the parameter in altitude 4500 m), and Hyp+SW-treated groups (rats exposed to hypoxia in hypobaric chamber and received increasing doses of Tsantan Sumtang treatment, 1.0 g/kg, 1.25 g/kg, and 1.5 g/kg). Upper panel: relative expression levels of p27Kip1 protein (optical density of p27Kip1 normalized against *β*-actin expression). Lower panel: *β*-actin protein expression was used as control. p27Kip1 expression was notably decreased in Hyp group and low dose of Tsantan Sumtang treatment rats, but the 1.25 g/kg and 1.5 g/kg of Tsantan Sumtang administration significantly elevated p27Kip1 protein expression and had no difference with the control rats (^Δ^*P*< 0.05 compared to Hyp; ^*∗*^*P*< 0.05 compared to Ctr).

The Western blot analysis showed that the cyclin D1 and CDK4 levels in the hypoxia group were higher than those in the control group (*P*< 0.05, Figures 6(a) and 6(b)). All 3 doses of Tsantan Sumtang significantly decreased the expression level of cyclin D1 compared to the hypoxia group, and the high dosage of medicine downregulated cyclin D1 expression to below the control group level. CDK4 expression was inhibited in the middle and high dosage groups (*P*< 0.05, Figure 6(b)), and expression in the high dosage group was not different from that in the control group (Figure 6(b)). In addition, the p27Kip1 levels in the hypoxia and low dosage groups were observably lower than that in the control group (*P*< 0.05, Figure 7). With the administration of Tsantan Sumtang, the middle and high dosage groups showed meaningful differences compared to the hypoxia rats, and there was no difference compared with the control rats, implying that these 2 dosages of Tsantan Sumtang could increase the p27Kip1 expression to a nearly normal level (Figure 7).

**Figure 8:**
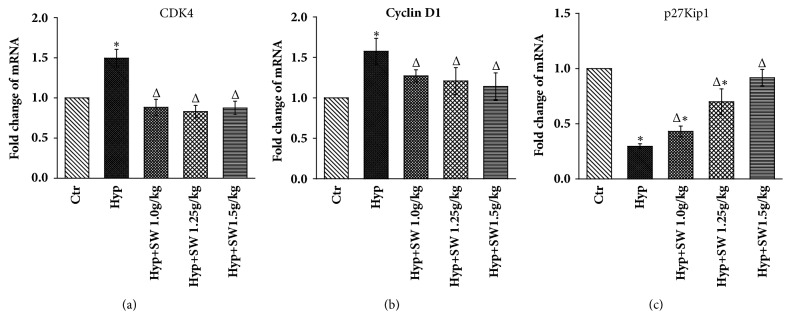
**Effects of Tsantan Sumtang on CDK4, Cyclin D1, and p27Kip1 mRNA expression by qRT-PCR in lung tissue of HPH rats (n=5).** Ctr: control group; Hyp: untreated hypoxia group (rats exposed to hypoxia in hypobaric chamber, equal to the parameter in altitude 4500 m), and Hyp+SW-treated groups (rats exposed to hypoxia in hypobaric chamber and received increasing doses of Tsantan Sumtang treatment, 1.0 g/kg, 1.25 g/kg, and 1.5 g/kg). *β*-actin gene expression was used as a control. Relative mRNA expression levels of CDK4, Cyclin D1, and p27Kip1 were shown. All 3 doses of Tsantan Sumtang meaningfully downregulated the CDK4 and Cyclin D1 gene expression compared to hypoxia groups and had no difference compared to control groups. Meanwhile, the p27Kip1 was increased significantly by Tsantan Sumtang, and the 1.5 g/kg dose showed no difference with the control group (^Δ^P< 0.05 versus untreated hypoxic rats; ^*∗*^*P*< 0.05 compared to Ctr).

In addition, the mRNA expression detected by RT-qPCR showed that all 3 doses of Tsantan Sumtang meaningfully downregulated CDK4 and cyclin D1 gene expression compared to the hypoxia group, and there was no difference compared to the control group (Figure 8). p27Kip1 expression was significantly increased by the 1.25 g/kg and 1.5 g/kg Tsantan Sumtang dosages, and expression with the 1.5 g/kg dosage was not different from that of the control group (Figure 8).

## 4. Discussion

It is well known that chronic hypoxia causes pulmonary vasoconstriction and pulmonary vascular cells proliferation, progressive right ventricular hypertrophy, and eventual right heart failure, and the major pathological changes of HPH are the pulmonary vasoconstriction and remodeling resulting from pulmonary artery smooth muscle cell (PASMC) proliferation [19–23]. The pharmacological prevention of HPH has focused on the modulation of PASMC proliferation, the activity of matrix metalloproteinases, and cell apoptosis. However, these medicines currently have limitations in clinical applications [6, 8].

Tsantan Sumtang, a Tibetan compound medicine, has been used for cardiovascular diseases in Qinghai and the Tibetan Plateau for years. The researches on effects and mechanism of components in Tsantan Sumtang on cardiovasculature showed that (+)-catechin, (+)-catechin-7-O-*β*-D-glucopyranoside from* C. axillaris *inhibited the proliferation of K562 cells and (+)-catechin also showed antihypoxia effect [24]. And the pretreatment with total flavonoids of* C. axillaris *(TFC) strongly improved cardiac function and obviously reduced heart pathologic lesion in ischemia/reperfusion (I/R) rat heart by increasing the levels of catalase, glutathione peroxidase, and superoxide dismutase in heart homogenate and decreasing that of malondialdehyde level [24, 25]. Research on phenolic acids in* C. axillaris* showed that gallic acid and protocatechuic acid showed obvious effect of antioxidant, antihypoxia, scavenging free radicals, and inhibiting platelet aggregation [26]. It is reported that myristyl ether and elemene in* M. fragrans* may obviously slow down the ventricular activity, which is consistent with its traditional usage of heart disease [27]. Lahlou et al. found that intravenous injection of methyl eugenol into mice could cause hypotension and the mechanism might be that methyl eugenol acts directly on the vascular smooth muscle to produce dilated blood vessels [28]. Our research findings of Tsantan Sumtang on HPH showed the consistence with some of the effects and mechanisms mentioned in the above researches. Indeed, our previous research found that Tsantan Sumtang could significantly decrease the rise in the mPAP of HPH model rats; however, the mechanism remained unclear [11]. Therefore, in this study, we evaluated the effect and mechanism of action of Tsantan Sumtang on pulmonary vasoconstriction and vascular remodeling in HPH rats. We found that Tsantan Sumtang induced the vasorelaxation of rat pulmonary arteries precontracted with NE in a concentration dependent manner* in vitro* while significantly reversing the pulmonary arterial wall thickening induced by chronic hypoxia* in vivo*. Histological observation also revealed that, with the Tsantan Sumtang treatment, there was an alteration in the dysplasia of the smooth muscle layer in the pulmonary arterioles.

Our previous and current study both found that Tsantan Sumtang could significantly decrease the rise in the mPAP of HPH model rats. Additionally, in this study, pulmonary artery ring perfusion* in vitro,* which simulated the vasoconstriction in HPH, proved the vasorelaxation effect of Tsantan Sumtang. Different inhibitors were used to clarify the mechanisms involved. It has been suggested that the NO-cGMP and PGI2-cAMP pathways are the major ways to adjust endothelium-dependent vascular relaxation [12]. For further investigation, we used 100 *μ*mol/L L-NAME, an inhibitor of NO integration into endothelial cells. The data showed that L-NAME considerably diminished the vasodilation resulting from Tsantan Sumtang treatment at the 0.9 and 1.2 mg/mL levels, signifying that NO plays a role in the vasorelaxation induced by Tsantan Sumtang, and the medicine improved the production of NO from L-arginine by endothelial nitric oxide synthase (eNOS) in the vascular endothelium and stimulated guanylate cyclase, thereby catalyzing the transformation of GTP to cGMP and reducing the internal Ca^2+^ flow through cGMP-dependent protein kinase [12]. Thus, the pulmonary arteries were dilated by increasing the intake of Ca^2+^ in the sarcoplasmic reticulum by Ca^2+^-ATPase or by directly leading to contractile protein dephosphorylation [29]. Furthermore, IMT, an inhibitor of cyclooxygenase (COX, a key PGI2 synthetase), had an even stronger influence at the same concentration levels, indicating that PGI2 was more likely to be involved in the vasorelaxation by Tsantan Sumtang and to play a more important role. This result also indicated that Tsantan Sumtang enhanced cAMP production through the effect on smooth muscle cells by means of PGI2 in vascular endothelial cells, inducing pulmonary vessel dilation through the PGI2-cAMP pathway. An interesting phenomenon was that both the vasorelaxation effect of Tsantan Sumtang and the inhibitory influences of 4-AP and IMT reached their maximum at a dose of 1.2 mg/mL compared to the other 3 doses from 0.3 to 0.9 mg/mL, which demonstrated the medicine performed its strongest vasodilation effect at its highest dose* in vitro*. Our previous drug safety evaluation research showed no evidence of toxicity, even in rats treated with 150 times the regular adult dosage for 90 days [11]. This result may give some guidance for the future use of Tsantan Sumtang in HPH patients.

It is generally accepted that an increase in intracellular Ca^2+^ initiates the contraction and proliferation of PASMCs and increases pulmonary vessel resistance; K^+^ channels regulate pulmonary vessel tone by cytoplasmic K^+^ and Ca^2+^ concentration and membrane potential [12]. The opening of K^+^ channels prompts the cell membrane to become hyperpolarized, resulting in the closing of L-type calcium channels and the decline of intracellular Ca^2+^ [30, 31]. Our research showed that BaCl_2_ (an inhibitor of K_IR_ channels), 4-AP (an inhibitor of Kv channels), and TEA (an inhibitor of BK_Ca_ channels) extraordinarily diminished the vasorelaxation effect of Tsantan Sumtang at all levels from 0.3 to 1.2 mg/mL, illustrating that the K_IR_, Kv, and BK_Ca_ channels were all engaged in such influence. In addition, the result showed that Tsantan Sumtang-induced vasorelaxation was considerably weaker at all 4 doses after exposure to BaCl_2_ and TEA compared to 4-AP (all at 1 mmol/L), and BaCl_2_ demonstrated strongest inhibitory effect at the 0.9 and 1.2 mg/mL levels. BK_Ca_ channels, with seven transmembrane domains and a calcium-binding zone (bowl), are fairly structurally distinctive, even among the K_Ca_ channel family [32–36]. BK_Ca_ channels have also been suggested as probable remedial targets for circulatory diseases based on their ability to adjust vascular tension [21, 33, 36].

Based on the above results, the PGI2-cAMP pathway, NO-cGMP pathway, and the opening of K^+^ channels (inward rectifier K^+^ channels, large conductance Ca^2+^-activated K^+^ channels, and voltage-dependent K^+^ channels) seemed to be the main factors in Tsantan Sumtang-induced concentration-dependent pulmonary artery vasorelaxation. For monomer vascular dilators, acetylcholine shows weaker maximum vasorelaxation influence via the NO-cGMP pathway, but echinacoside is a very similar dilator at its highest concentration through the opening of NO-cGMP-PKG-BK_Ca_ channels [37, 38]. According to these reports, both Tsantan Sumtang and monomers exhibited concentration-dependent vasorelaxation effects and reached their maximum rates at one specific dose, while as a compound medicine, the role of Tsantan Sumtang in vasorelaxation occurs through a combination of more pathways than that of monomers; however, further research is still needed [37, 38].

We successfully developed the animal model of HPH by using an automatically adjusted low-pressure hypobaric chamber. The mPAP and RVHI of the HPH model rats had an increased average of 16.92 mmHg and 14.14%, respectively, compared with the control group (*P< 0.05*, Figures 3(a) and 3(b)). With the treatment of Tsantan Sumtang, both the mPAP and RVHI decreased significantly in comparison with the hypoxia group (*P< 0.05*, Figures 3(a) and 3(b)). However, as HPH is defined by mPAP ≥ 25 mmHg at rest and right ventricular hypertrophy is a compensated outcome from the sustained increase of the mPAP in HPH, we propose that the medicine significantly decreased the mPAP while having a protective influence regarding right ventricular hypertrophy [39, 40].

Regarding the morphometric experiments* in vivo*, the thickening and ultrastructural change of the pulmonary artery wall proved the successful establishment of the HPH model rats (Figure 4). Histological observations revealed that the treatment with Tsantan Sumtang greatly corrected hyperplasia of the smooth muscle layer in the pulmonary arterioles, and the WT%, as the percentage of vascular wall thickness, was obviously decreased compared to the HPH model rats (*P< 0.05*, Figure 4). As the above results showed that Tsantan Sumtang significantly improved HPH, we further explored the proliferation related protein expression levels of PCNA and *α*-SMA by Western Blot analysis.

PCNA, one of the molecular markers of cell proliferation that is mainly detected in the S phase of the cell cycle, is a nuclear protein and DNA polymerase cofactor, and resting cells hardly express PCNA [41, 42]. As a subtype of muscle fibrocytes and a molecular marker of activated myofibroblasts, the presence of *α*-SMA indicates the transition of cells to the myofibroblast phenotype [43]. Compared to the control group, the high expression levels of PCNA and *α*-SMA shown by the Western blot analysis indicated that PASMC proliferation occurred in the HPH model rats. The results also revealed that Tsantan Sumtang significantly reduced the hypoxia-induced high protein expression levels of PCNA and *α*-SMA in the pulmonary artery (*P*< 0.05, Figure 5), which was in accord with the morphological results shown by HE staining and TEM, especially the alleviation of the tunica media thickening in the pulmonary arterial vascular wall as well as the decreased WT% data previously discussed (Figure 4).

To elucidate the antiproliferative mechanism of Tsantan Sumtang observed in Figures 4 and 5, the protein and gene expression of CDK4, cyclin D1, and p27Kip1 were detected by Western Blot analysis and qRT-PCR, respectively. CDK4 and cyclin D1 are key regulators in the switch from the G_0_/G_1_ phase to the S phase during cell proliferation. As cyclin-Cdk complexes, these regulators activate important transcription factors in cell cycle progression. p27Kip1, as the key CDK inhibitor, plays major roles in cell-cycle regulation. p27Kip1 binds cyclin-Cdk multiplexes and inhibits the hyperphosphorylation of the retinoblastoma protein, causes G_1_ blockage, and limits cell proliferation [44, 45]. This result is in accordance with other studies and shows that p27Kip1 acted as a negative regulator of cell proliferation by downregulating the expression of CDK4 and cyclin D1 [16–18, 45, 46]. Compared with the hypoxia group, the CDK4 and cyclin D1 levels in the Tsantan Sumtang groups were distinctly decreased. The above results combined to illustrate the underlying mechanisms might be involved in the antiproliferation and antiremodeling effect of Tsantan Sumtang on pulmonary arterioles (Figures 5, 6, and 7).

Our study found that the opening of K^+^ channels was greatly involved in the dilation induced by the medicine and that the decreased p27Kip1 expression in the hypoxia group was elevated with Tsantan Sumtang administration, which might demonstrate the underlying mechanisms involved in the effect of Tsantan Sumtang against HPH. Accordingly, we may presume that Tsantan Sumtang significantly improved HPH in the rats by reducing hypoxic pulmonary vasoconstriction and diminishing PASMC proliferation through decreasing the expression levels of CDK4 and cyclin D1 with the elevation of their negative regulator p27Kip1.

## 5. Conclusions

This study explored new effects and the underlying mechanisms of Tsantan Sumtang on HPH. Our research showed that Tsantan Sumtang could be a potential preventative medication for HPH, in addition to its traditional cardiovascular usage. We found that the administration of Tsantan Sumtang prevented HPH based on pulmonary artery vasorelaxation and the reversal of proliferation. The mechanism involved in pulmonary artery vasorelaxation might be related to the PGI2-cAMP pathway, NO-cGMP pathway, and the opening of K^+^ channels (inward rectifier K^+^ channels, large conductance Ca^2+^-activated K^+^ channels, and voltage-dependent K^+^ channels). Tsantan Sumtang also alleviated pulmonary vascular cells proliferation in HPH rats by suppressing cyclin D1 and CDK4 expression through the inhibition of p27Kip1 degradation. Further research of effects and the corresponding mechanisms of Tsantan Sumtang related to HPH is needed and will be ongoing.

## Data Availability

The research is funded by the National Natural Science Foundation of China (No. 81660308), www.nsfc.gov.cn, and Ministry of Human Resources and Social Security of China (No. 386,522), www.mohrss.gov.cn, and still has closely related experiments ongoing, so the data are not available right now but could be reached by contacting the corresponding author Dianxiang Lu at ludianxiang@qhu.edu.cn after 2021.
